# An approach to measuring and encouraging research translation and research impact

**DOI:** 10.1186/s12961-016-0131-2

**Published:** 2016-08-09

**Authors:** Andrew Searles, Chris Doran, John Attia, Darryl Knight, John Wiggers, Simon Deeming, Joerg Mattes, Brad Webb, Steve Hannan, Rod Ling, Kim Edmunds, Penny Reeves, Michael Nilsson

**Affiliations:** 1 Hunter Medical Research Institute (HMRI) Lot 1, Kookaburra Circuit, New Lambton Heights, Newcastle, NSW 2305 Australia; 2 School of Medicine and Public Health, The University of Newcastle, University Drive, Callaghan, NSW 2308 Australia; 3 Department of Medicine, John Hunter Hospital, Hunter New England Local Health District, New Lambton Heights, NSW 2305 Australia; 4 Edith Cowan University, 270 Joondalup Drive, Joondalup, WA 6027 Australia; 5 The University of Newcastle, University Drive, Callaghan, NSW 2308 Australia; 6 Experimental & Translational Respiratory Medicine, Priority Research Centre for Asthma and Respiratory Diseases, Hunter Medical Research Institute & University of Newcastle, Newcastle, NSW Australia; 7 Department of Respiratory and Sleep Medicine, John Hunter Children’s Hospital, Newcastle, NSW Australia; 8 Central Queensland University, Brisbane, 4000 Australia; 9 School of Biomedical Science and Pharmacy, The University of Newcastle, University Drive, Callaghan, NSW 2308 Australia

**Keywords:** Research impact, Research translation, Outcome research, Outcome measurement, Evaluation, Performance monitoring and feedback

## Abstract

**Background:**

Research translation, particularly in the biomedical area, is often discussed but there are few methods that are routinely used to measure it or its impact. Of the impact measurement methods that are used, most aim to provide accountability – to measure and explain what was generated as a consequence of funding research. This case study reports on the development of a novel, conceptual framework that goes beyond measurement. The Framework To Assess the Impact from Translational health research, or FAIT, is a platform designed to prospectively measure and encourage research translation and research impact. A key assumption underpinning FAIT is that research translation is a prerequisite for research impact.

**Methods:**

The research impact literature was mined to understand the range of existing frameworks and techniques employed to measure and encourage research translation and research impact. This review provided insights for the development of a FAIT prototype. A Steering Committee oversaw the project and provided the feedback that was used to refine FAIT.

**Results:**

The outcome of the case study was the conceptual framework, FAIT, which is based on a modified program logic model and a hybrid of three proven methodologies for measuring research impact, namely a modified Payback method, social return on investment, and case studies or narratives of the process by which research translates and generates impact.

**Conclusion:**

As funders increasingly seek to understand the return on their research investments, the routine measurement of research translation and research impact is likely to become mandatory rather than optional. Measurement of research impact on its own is insufficient. There should also be a mechanism attached to measurement that encourages research translation and impact – FAIT was designed for this task.

## Background

It is an unfortunate reality that a substantial amount of effective research does not translate, is not implemented and does not create impact [1]. Grimshaw et al. [2] and others note that, despite extensive investments in research and development, relevant research findings are not being fully implemented by healthcare systems and are not being appropriately used by others in the chain of scientific research [2–8]. The implication from sub-optimal levels of research translation is that the return on research investments is also lower than it could potentially be. Despite awareness of this problem, the misalignment between the generation of research outcomes and the use, or application, of those outcomes is not being adequately addressed [9].

This study reports on the development of a framework, referred to as the Framework to Assess the Impact from Translational health research (FAIT), designed to both measure and encourage research translation and research impact. The novelty of this framework, and what it adds to the impact measurement field, is that it is designed to do more than provide a mechanism for demonstrating accountability from funded research, which is often the aim of impact measurement frameworks. Our framework was designed with the explicit aim of encouraging specific activities and behaviours associated with research translation. The underlying assumption is that research translation is a prerequisite for future research impact. The impetus for developing this framework, by researchers affiliated with the Hunter Medical Research Institute (HMRI), stemmed from an aspiration to both demonstrate and optimise research translation and impact.

We use two terms throughout this article: ‘research translation’ and ‘research impact’ and have defined how we use them.

### Research translation

We developed this working definition because there are many terms in the literature to define the process of translating research-generated knowledge to others [10–13]. Our working definition is:

…Research translation is a process of knowledge generation and transfer that enables those utilising the developed knowledge to apply it. This definition acknowledges that, once generated, knowledge flows can be multidirectional and non-sequential.

This definition recognises four core aspects of research translation that FAIT would need to support. Firstly, that research translation involves a stage of knowledge generation, for example, new insights generated from clinical trials. Secondly, that it requires the generated knowledge to be passed-on or shared. Thirdly, that knowledge sharing provides an opportunity to apply the new information. Finally, that the flow of knowledge is multidirectional and non-sequential.

### Research impact

As with ‘research translation’ the literature contains many meanings of the term ‘research impact’ [14]. The working definition for ‘research impact’, tailored for health and medical research, is:

…the demonstrable effect from the flows of knowledge between basic, patient and population-orientated research, and clinical trials, that improves human health and quality of life, and generates benefits for the economy, society, culture, national security, public policy, or the environment.

This definition recognises the contributions made across the science spectrum and places prominence on human health and quality of life. It includes flow-on effects, such as increased productivity, reduced waste, and contributions to economic growth, as well as traditional academic outcomes. The measurement of what is herein referred to as ‘research impact’ is based on this definition.

The objectives guiding this case study were to (1) use the existing literature to understand the range of measurement frameworks and techniques relevant to measuring and encouraging research impact; (2) design a prototype framework capable of measuring and encouraging both research translation and research impact; and (3) to refine the framework with input from a Steering Committee.

## Methods

This study was a pragmatic response by researchers at HMRI to tackle the disparity between the creation of research outputs and the uptake of those outputs. As an organisation, HMRI has a number of attributes which influenced this study; firstly, HMRI researchers work across the spectrum of health and medical research from basic to applied science. Secondly, the organisation participates in research across a diverse range of diseases and health services. Thirdly, the organisation has significant engagement with a patient communities, heath policymakers and the healthcare industry as well as academic and clinical researchers. Finally, HMRI is a facilitator of health and medical research as well as being a funder of this research.

The study was based on a mixed methods approach and involved the following: (1) a scoping review of existing research impact frameworks and techniques, which served as the basis for the development of FAIT; (2) a development stage to design the prototype of FAIT; and (3) a feedback stage where iterations of the evolving FAIT were presented to our Steering Committee with the aim of eliciting views and suggestions on how it could be improved.

### Objective 1 – Use the existing literature to understand the range of measurement frameworks and techniques that are relevant to measuring and encouraging research translation and research impact

The objective of this scoping review was to identify the range of frameworks and techniques used to measure and encourage research translation and impact. This literature was then used as a platform to develop a prototype framework to measure and encourage research impact.

The literature searches were conducted using NEWCAT+, available through the University of Newcastle library and limited to full text availability in English. This search tool accesses journal articles listed in electronic databases including OVID, Science Direct, Medline and Econlit. Further literature searches were conducted using references and citations from relevant papers. The search was also conducted in Google scholar and Google to identify literature from government departments, international organisations and research funders with an interest in the measurement of research translation and/or research impact. These sites included the Australian Research Council, National Health and Medical Research Council, and WHO.

Search terms included ‘measuring research + (impact or outcomes)’, ‘economic impact + (research or basic science or applied science)’, and ‘measuring research impact’. From the identified literature, further source materials were identified using relevant references. Additional references were also provided by reviewers of the original version of this paper.

Impact assessment methods identified in the literature were considered for our framework if they addressed one or more of the following criteria: (1) generated a result from measurement that would have meaning to research funders; (2) facilitated communication of complex translational processes; and (3) encouraged, or had the potential to encourage, research translation and research impact.

### Objective 2 – Framework design and development

The design of FAIT was mostly conducted by health economists at HMRI who examined the frameworks and techniques found in the literature. The usefulness of this information was guided by the aims guiding the design of FAIT. These were to (1) capture processes, outcomes and impacts generated across the spectrum of health research from discovery to applied science; (2) encourage research translation; (3) enable the implementation of improvement processes when research translation fails; (4) utilise cost-effective data collection techniques; and (5) facilitate communication on research impact. Non-economists were also involved in the design and development of FAIT and were part of the Steering Committee overseeing FAIT’s refinement.

### Objective 3 – Framework refinement

Once the prototype framework was developed, stakeholder feedback was collected from a project Steering Committee whose membership represented research funders, clinicians, basic science researchers, applied science researchers, primary healthcare, and university administration. Steering Committee members are included in our authorship (AS, JA, DK, JW, JM, BW, SH and MN).

The process involved successive presentations of FAIT, in its various stages of development, to the Steering Committee over 2014 and 2015. Feedback from each presentation was considered by the economist authors (AS and SD) and used, where appropriate, to refine FAIT. This attempt at co-design aimed to ensure the prototype of FAIT reflected the needs of a broad range of end users. A national presentation on FAIT was also made at the Australian National Health and Medical Research Council’s Symposium on Research Translation [15]. Comments received by the authors as a result of this presentation were also included in the design.

## Results

### Objective 1 – The range of measurement frameworks and techniques relevant to the measurement and encouragement of research translation and research impact

As numerous reviews have been conducted on frameworks to measure research impact, our intention was not to conduct another appraisal of this literature. Instead, our goal was to use the existing reviews to understand the range of frameworks and the techniques available to measure research translation and research impact. As FAIT was to be designed to both measure and encourage research translation and research impact, additional techniques were also considered if they had a potential to assist the ‘encouragement’ aspect of FAIT’s design. A summary of information extracted from influencing articles is provided below.

The systematic review by Banzi et al. [16] and a more recent review by Milat et al. [17] provided the basis for understanding the range of frameworks and techniques from measuring research impact. More recent expansion on this literature is provided by Greenhalgh et al. [18], who report on existing methods to measure research impact as well techniques that are under development.

The measurement of research impact is a relatively new field, and while the methods have been developing since the 1990s [19], most activity in this space has taken place since 2006 [17]. Banzi et al. [16] identified broad categories of frameworks based on bibliometrics, econometrics and ad hoc case studies. When considering all available frameworks, the most frequently used method was Payback, a finding confirmed by Banzi [16], Milat [17] and Greenhalgh [18].

The study presented here focuses on three measurement methods: Payback, economic evaluations, and case studies. These methods meet most of the acceptance criteria for this case study and they cover a broad spectrum of impact assessment techniques, particularly because Payback, or a derivation of Payback, is the basis of many other impact measurement frameworks [18].

#### Payback

The Payback Framework, developed in the 1990s by British researchers Buxton and Hanney [20], is the most common method employed for measuring research impact [15]. The method is based on the identification of domains of benefit such as knowledge impacts, research impacts, and political and policy impacts. Payback is usually implemented through semi-structured interviews to obtain the perspective of researchers as to the impact of their research [17]. This information is supported by bibliometric analysis and verification studies [17]. The technique provides a scorecard on the payback to society for investing in research and it is widely used in Australia, the United Kingdom and Canada [16, 20–26]. Conceptually, Payback can be modified to be the basis of a prospective measurement framework. It can also be modified to populate the domains of benefit with quantitative metrics [16], rather than qualitative interview data.

The Payback method is intuitive and the results provide a sense of the outputs and outcomes produced in broad domains relevant to policymakers, funders and the general community. However, while the Payback methodology is well developed, it requires substantial resources to implement. The labour intensity of implementing Payback is a consequence of the combination of researcher interviews, document analysis and validation work that feeds into the assessment. If measurement becomes a disproportionate burden to the activity being measured, there is a real risk it will not be undertaken. This is one possible reason why the routine measurement of research impact remains elusive [13]. It is for this reason that some evaluators have modified the Payback Framework to reduce resource intensity [18].

#### Economic measures

Economic measures for evaluation typically compare cost against a measure of outcome; they often report outcome measures expressed in monetary terms or rates of return. For the purpose of measuring research translation and research impact, it is preferable that multiple benefits be included. For this reason, out of the suite of potential economic evaluation techniques, cost-benefit analysis (CBA) stood out as being the appropriate tool. Further, as costs and consequence are financial values, the reportable metric of CBA is a ratio of benefit per dollar of cost, or a ‘return on investment’. CBA is the basis of a more encompassing Social Return On Investment (SROI) analysis, which takes a broader perspective of the range of benefits captured and reported [27]. SROI is an appealing technique for evaluating health-related research because it allows the inclusion of flow-on impacts from improved patient health such as improved worker productivity. From a societal viewpoint, SROI reports the return on investment where benefits include public and private returns.

Although interesting on many levels, the economic approaches have drawbacks. One of these is that economic modelling is frequently based upon simplifying assumptions [18], such as time lags between discovery and utilisation, extent of uptake, and contentious monetised values placed on some benefits. An example of the latter would be a financial value applied to a given improvement in ‘quality of life’. Despite these drawbacks, economic approaches have been used in Australia to provide estimates of the benefit from investments into health and medical research [28–30] based on top-down modelling approaches. These analyses made extensive assumptions to estimate both attribution to a body of research and the estimated benefits [18]. Bottom-up approaches can alleviate the attribution problem when, for example, cost, cost-effectiveness, cost-utility, and cost-benefit analyses are derived from data collected from controlled trials. These data allow the evaluation to be more precise in relating attribution to a new intervention [18].

#### Case studies

The United Kingdom RAND organisation reviewed selected frameworks to measure research impact and determined that evidence-based case studies for measuring impact were superior to quantitative metrics, even when the case studies were prepared by the research leaders being evaluated [31]. The strength of case studies is that they provide a narrative of the often complex and bidirectional knowledge flows. While case studies may rely on expert advisory panels to review qualitative impact statements [31], they are prone to the same biases that characterise self-reports such as selective memory [16] and, importantly, they cannot produce the quantitatively based metric of ‘return on investment’. Further, the construction of case studies tends to be resource intensive, reducing their viability for widespread and routine measure of research impact [16]. Nonetheless, case studies provide worthwhile contributions particularly by describing the often complex pathways for research translation. These descriptions can be powerful tools for communicating the nature and extent of research translation and, ultimately, research impact.

#### Other measurement approaches

Of the identified, alternative techniques for measuring research impact, many were found to be modified or adapted forms of Payback. For example, the Canadian Academy of Health Sciences framework and the Research Impact Framework; the latter was designed as a checklist to encourage consideration of translational processes [18]. Technological advancements in electronic databases have also opened new avenues to collect and report data that can be used to conduct an impact assessment [18]. For example, commercially available electronic services (such as Researchfish^®^) collect data on publications, citations and other ‘macro’ sources and then combines this information with uploaded ‘micro’ data from research teams on collaborations, prizes, and other outputs and outcomes [18].

##### Program logic models

The construction of a program logic model is often viewed as a formative methodological step in measuring research impact [18, 32]. They are not measurement frameworks on their own, but they provide the rationale linking the research aims and activities to research outputs, and from research outputs through to anticipated impact [18, 33]. In their standard form, these models provide a conceptual linkage between inputs, activities, outputs and impact [18]. Typically, these models are predictive; anticipating the outputs and outcomes from research-generated knowledge.

In their basic form, the linear nature of program logic models has been criticised as providing an over simplification of the complex pathways observed in the lifecycle of research development, transfer, utilisation and, ultimately, impact [34]. A more fluid, and less linear, approach would allow feedback loops between the different actors in the research translation pathway [34]. It is also argued that any predictive powers of program logic models will be weakened if the research findings are unclear or simply identify the extent of the problem, rather than providing an evidence-based solution [34].

##### Performance frameworks

Performance frameworks were not typically found amongst the reviews of impact measurement frameworks. While common reasons for measuring research impact tend to focus on accountability and advocacy [35–38], it is rare to find an impact measurement framework where the purpose of measurement is to explicitly encourage certain behaviours or activities. Insights from the quality improvement literature suggest a reason why research impact frameworks could consider aspects of performance monitoring and feedback. The use of continuous quality improvement in health and other fields, provides an evidence base for how it can modify performance. A Cochrane review on this issue found positive effects through an ‘audit and feedback’ mechanism to improve the performance of health professionals [39]. However, to improve performance or change behaviours, measurement needs to occur within a framework that links metrics to the process of improvement [40–42]. A cautionary warning is that performance monitoring requires careful metric selection to ensure the selected incentives drive the anticipated behaviour [43].

There are arguments against using such an approach because of its resemblance to a prescriptive specification sheet. Ward [44] argues a checklist of translational activities is a sub-optimal approach to increasing research translation as its content would not reflect the complex and multidirectional processes by which research translates. A more productive approach, would be for researchers to understand the complexity of translational process so that they can embed appropriate and tailored translational interventions into their research [44].

### Objective 2 – Framework design and development: a conceptual model for measuring and encouraging research translation and research impact

FAIT was the product of understanding the components of research impact frameworks and was designed to address the aims of (1) capturing processes, outcomes and impacts generated across the spectrum of health research from discovery to applied science; (2) encouraging research translation; (3) enabling the implementation of improvement processes when research translation fails; (4) utilising cost-effective data collection techniques; and (5) facilitating communication on research impact.

As a consequence of the ‘encouragement’ aim, FAIT is designed to be prospective tool, implemented at the start of a research program. FAIT is based on a modified program logic model that guides the overall assessment, three core methods (a modified Payback approach, SROI and case studies), and uses a scorecard to report results.

#### A modified program logic model

The modified program logic model identifies (1) the need being addressed by the research program; (2) the research activities being supplied to meet the ‘need’; (3) the expected research outputs; (4) the end-users of those research outputs; and (5) the anticipated impact from the use of the research outputs. An advantage of this modification is that it identifies who will use the research products, i.e. the ‘end-user’. For basic research, the end-user might be other basic scientists or pharmaceutical companies interested in progressing the research, and for population health research, the end-user might be public health authorities.

The program logic model also can be used to tease out anticipated research outputs (e.g. new guidelines). Finally, it provides a view on how the research is anticipated to generate impact – and the types of impact that are expected. In our view, this information can guide the selection of impact metrics.

**Fig. 1:**
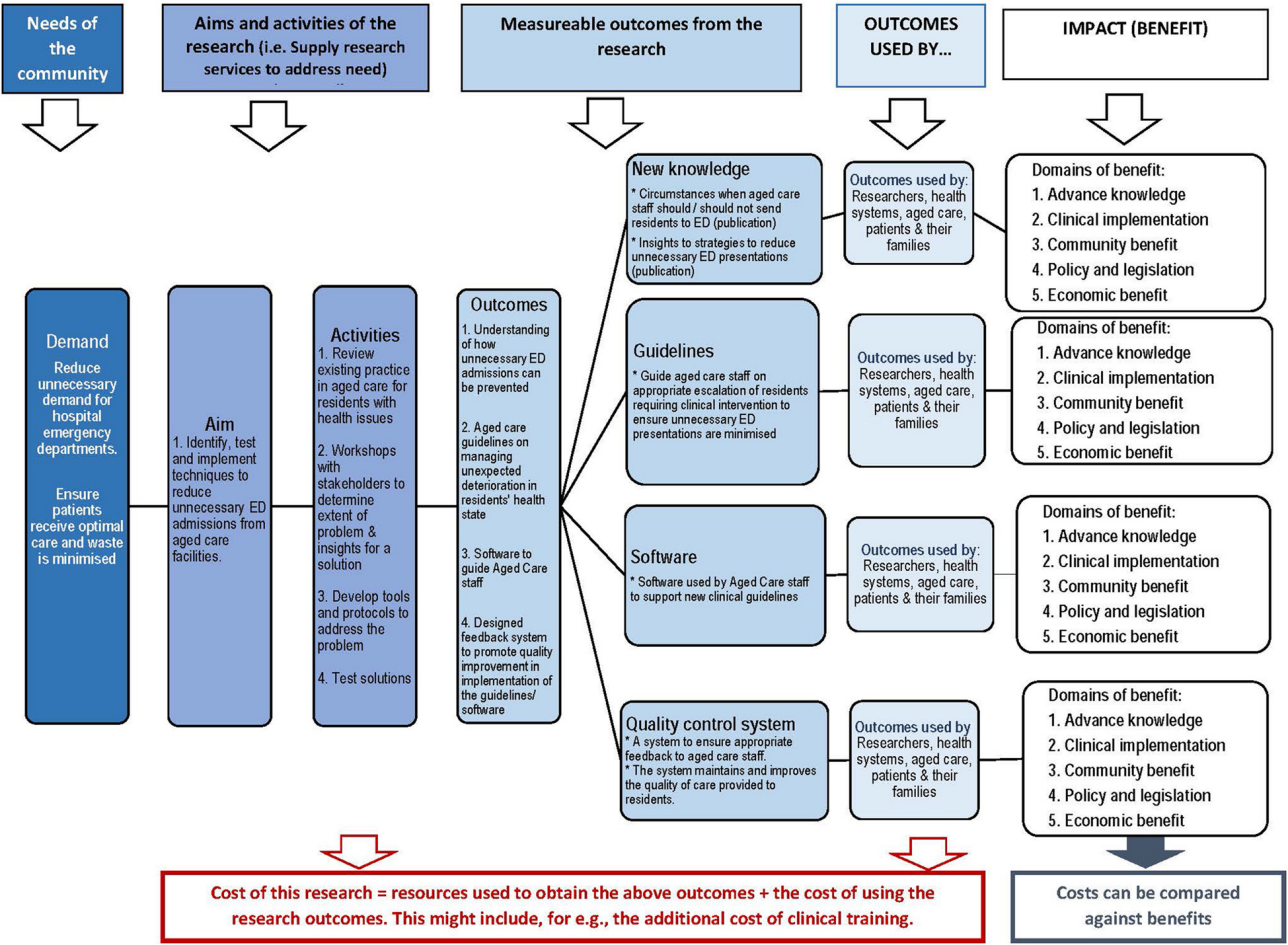
Hypothetical example of a logic map for research addressing unnecessary emergency department presentations from residents in aged care facilities

We accept that linear program logic models fail to replicate the complexity and ambiguities of the ‘research to utilisation’ cycle. However, the models are meant to be an approximation of the anticipated path for research translation and subsequent impact, i.e. it is a guide rather than a replica of reality. Further, the construction of the program logic model at the beginning of the research program allows for incremental modifications to its design over the life of the research program. That is, the program logic model can change over time, as unexpected research outputs become evident, political influence is exerted or, for other reasons, the research takes a different path to that originally planned. Figure 1 presents an example of a program logic model for an initiative to reduce unnecessary emergency department admissions from aged care facilities. The model links community need for the intervention to the research services that are being supplied in response to that need. The logic model identifies research products and the end-users who are expected to utilise these products. The model also provides the range of anticipated impacts from the research.

#### Three core methods incorporated into FAIT

While the program logic model provides an explanation of the linkages from knowledge generation to utilisation, three component methodologies provide the evidence to demonstrate research translation and help quantify research impact: a modified Payback approach, SROI and case studies.

Modifications to payback

1. *Domains of benefit*
  The first modification to Payback is to use the domains of benefit recognised by the Becker List [45] because of their inclusion of the domain of ‘Clinical implementation’, which is particularly relevant to health and medical research. These domains of benefit are shown in the far right hand column in Fig. 1.
2. *Determine relevant metrics for measuring impact*
  The second modification is to incorporate metrics to represent anticipated benefit or impact. These will include universal measures that will be applicable across the research spectrum, for example, metrics for Advancement of Knowledge might include publications and completed PhDs. Customised metrics will also be included that are tailored to the research program. Using metrics within each domain is similar to that proposed by Banzi et al. [16, 17] and initial work on possible metrics has been published [45]. These metrics, combined with prospective data collection, should minimise the need for expensive and potentially less accurate retrospective data collection. Some of the metrics will be structured to support the planned economic analysis.
3. *Inclusion of process metrics*
  The third modification to Payback is to include a module of process metrics – these are based on performance monitoring and feedback principles, and are separate to the measures of impact. They are designed to provide regular feedback to research leaders on key activities related to their research, including activities associated with research translation. Feedback allows research leaders/managers to assess whether the implementation of these activities is appropriate or whether they require attention. The potential is to use process metrics that support research translation activities and behaviours so that (1) they are identified to researchers and (2) their use is encouraged.
  There is a developing body of work as what these activities and behaviours might be [46–48]. For example, activities such as early engagement with end-users, the development of a strategic plan explaining the translational pathway (e.g. a program logic model), and an understanding of the barriers to translation, have been shown to be associated with successful research translation [2, 48]. Oliver et al. [47] provide a list of facilitators and barriers to research translation that could also be used as a starting point. Others have investigated the length of time research takes to translate and identified factors that may be associated with accelerated uptake. Hanney et al. [46] reported on the success of economic incentives to get drugs to market once Phase III trials were complete (and had demonstrated success). We see the process metrics as having flexibility so that researchers can also nominate other translational activities that might be tailored to their research.

##### SROI

The second method adopted by FAIT is based on an economic measure: SROI. The reporting of SROI provides useful information on the return received by society for investments into health-related research. This simple ratio is expressed as the number of dollars of community benefit per dollar of cost; it is well understood by policymakers, research funders and the broader community and allows direct comparison with SROI calculated for other programs. SROI is a metric that can be calculated using simulations, or projections, at the planning stage. These simulations can then be compared to subsequent recalculations using actual data as the research program progresses and delivers outputs.

The inclusion of project-based economic assessment methods, such as SROI, has several benefits. First, the methodical process of reviewing the research intervention, the anticipated pathways to impact, and determination of where and for whom costs and benefits accrue, serves to emphasise the key risks that need to be managed to realise the anticipated benefits, for example, to ensure effective implementation. The results of an economic assessment can also enable comparison between alternative interventions and provide evidence to address potential budgetary hurdles to impact. In contrast to top-down assessments, bottom-up economic analyses provide greater project-specific detail and, consequently, greater potential influence upon research activity. Economic analyses that express the result as a simple and widely understood ratio of ‘dollars of benefit per dollar of cost’, can also provide a compelling supportive argument to policymakers and the wider community.

##### Case studies

FAIT’s third method is based on case studies selected from the program of research. This introduces a qualitative aspect to the measurement of research impact and provided a narrative of how translation occurred and how research impact was generated. The case studies are expected to be supported with evidence extracted from the modified Payback and SROI. Case studies enable quantitative findings to be placed in context, and they are an opportunity to explain variances in research costs, outputs and impacts.

#### Reporting the results: a scorecard

**Fig. 2:**
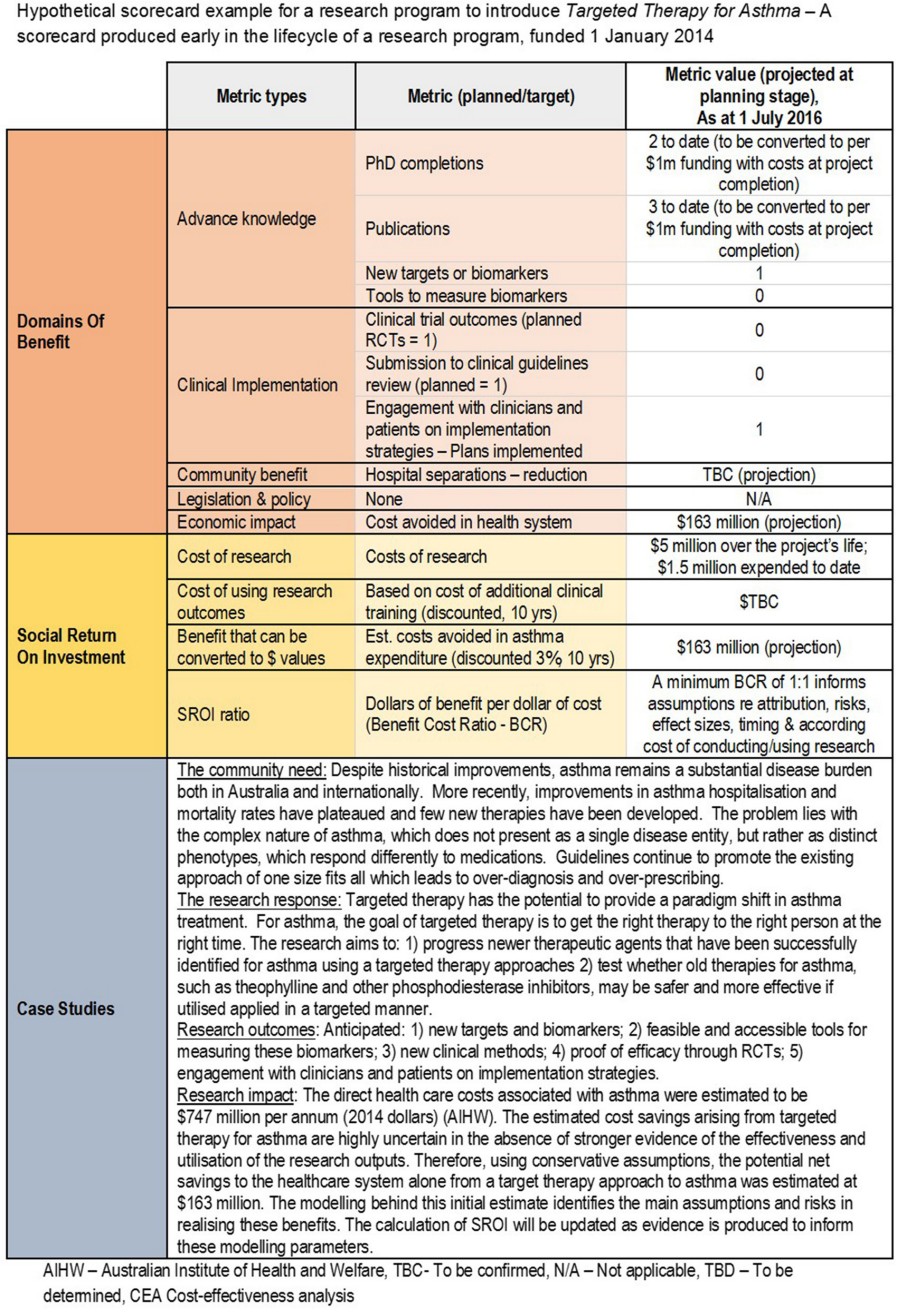
Hypothetical scorecard example – early stage basic science research

**Fig. 3:**
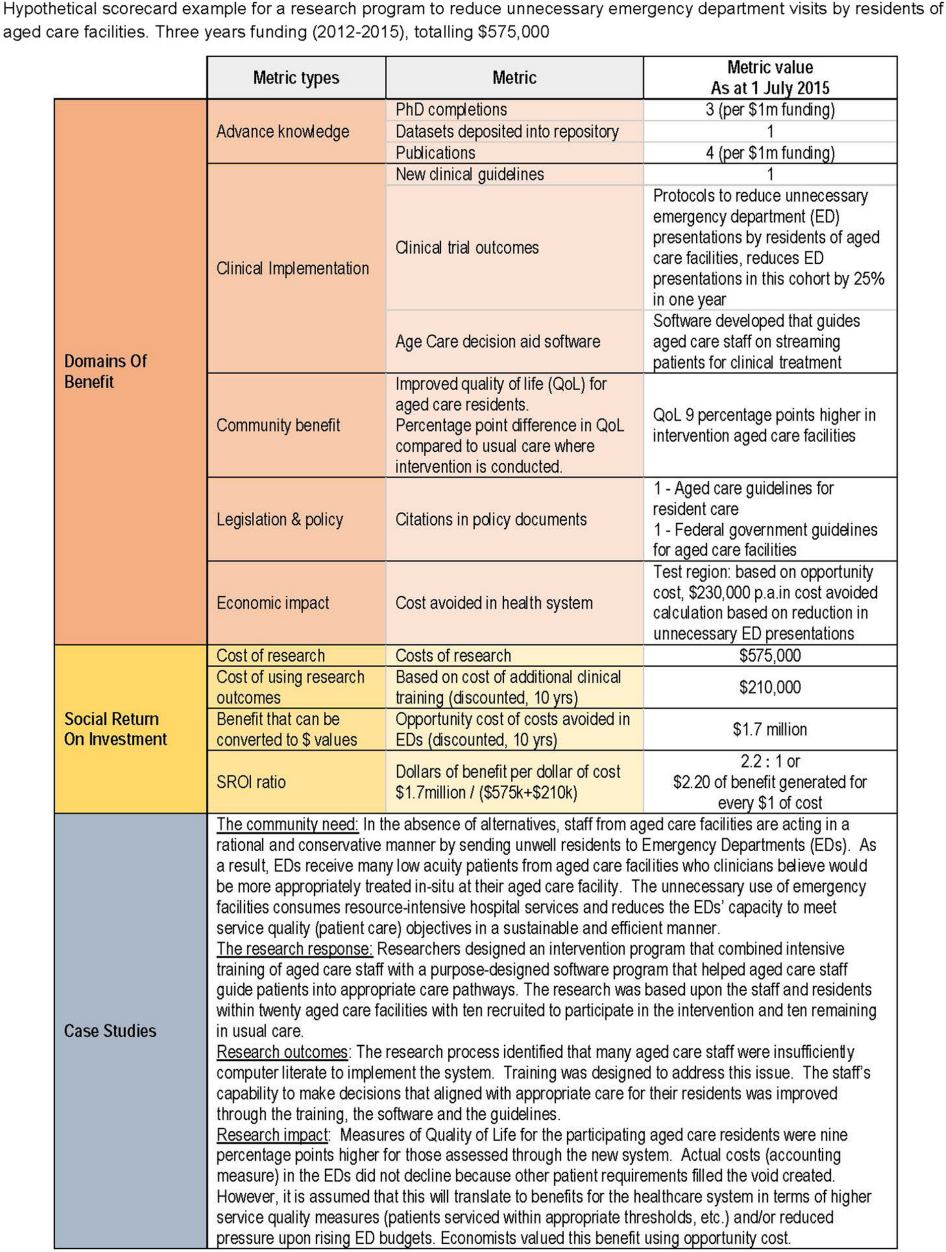
Hypothetical scorecard example – at completion of a clinical application of a model of care

Combining the outcomes from these three methods provides a scorecard to report research impact; the content of the scorecard grows as the research progresses. The scorecard (Figs. 2 and 3) is a summary of the outcomes from the three methods and allows the outcomes to be triangulated, strengthening the level of confidence in claimed research impact. The content of the scorecard will reflect where the research sits in its lifecycle. Newly established research will have little to report, while completed research would be expected to have a more complete scorecard.

The scorecard is designed to be a simple communication tool that contains top-level results for each of the three component methods. To report results, FAIT will express findings, where appropriate, with a common denominator such as completed PhDs per $1 m of funding. In practice, this will require a standardised research cost base, based on the cost of undertaking research within a specific country or geographic location.

The scorecard examples are taken from hypothetical basic science and applied science situations. Figure 2 shows the scorecard for research into targeted therapy for asthma patients. This scorecard represents an incomplete research program, early in its lifecycle, hence some benefits and costs are ‘to be confirmed’. Projections have identified potential cost savings to the health system, but there is more information to be collected over the course of the research to complete this scorecard. Figure 3 shows as example of implementing a model of care in a study population. At the point of providing this hypothetical scorecard, the research has been shown to be effective in the study population. It also shows the results from the three component methodologies.

### Objective 3 – Stakeholder inputs to FAIT

Stakeholder feedback was provided via our Steering Committee. A number of issues with the early prototype versions of the framework were identified. Stakeholders focused on potential bias in measuring impact, potential bias in the reporting of impact from different sized research projects and how the measurement framework would assist communication about the demonstration of research impact. Another issue concerned the cost of implementing FAIT.

First, stakeholders noted that the measurement of impact could create a bias against research that has an extended time between a research discovery being translated through to the point of use. This concern was addressed in two ways. First, the concerns reinforced the appropriateness of a broad working definition for research impact. A broad definition includes the consequence of research that may not have a patient-level impact, but generates an impact elsewhere such as contributing to a body of knowledge. Second, SROI supports scenario modelling, where the evidence available at given time points is used to model future states with and without (i.e. the counterfactual) specific research innovations. As new evidence becomes available, the key assumptions underpinning these modelled scenarios are adjusted. In Australia, a similar scenario modelling exercise is already required by one major research funder [49].

A further concern was how a research project’s size would possibly influence the communication of research impact. Larger research projects would be likely to have more opportunities to generate impact (e.g. more capacity opportunities through PhDs and post-doctoral positions) the expectation was that they would appear more successful than smaller research projects purely based on funding. FAIT addresses this concern by reporting, where feasible, the results as a function of a common standardised denominator, for example, PhDs completed per $1 m of funding.

‘Communication’ was raised in the context of the benefit of FAIT to researchers in providing a research translation and research impact narrative. This issue focused on the ability of FAIT to meet accountability objectives and assist researchers to demonstrate research translation and research impact, which are increasingly required for funding applications. Demonstration of these concepts is a core component of FAIT. This issue highlighted the need for a plainly written narrative explaining the need the research addresses, what was produced, and what impact was generated. Hence, the inclusion of case studies is an important communication mechanism.

The cost of implementing FAIT is yet to be determined. However, two Australian Centres of Research Excellence, with funding to 2019, have committed to implementing FAIT and this exercise will shed light on the resources required for implementation.

## Discussion

The purpose of this study was to develop a research impact framework designed to measure and encourage research translation and research impact. With a set of clear goals, an understanding of the strengths and limitations of existing impact frameworks, FAIT was developed and refined with the input of stakeholders. This novel framework explicitly encourages activities that are associated with research translation. It does this by including performance monitoring and feedback that targets activities and behaviours associated with research translation, with an underlying assumption that successful research translation is a forerunner of research impact.

Hence, the main strength in the application of FAIT is not just to report the outcomes from funded research but also to actively encourage researchers to consider research translation activities. The body of evidence as to what these activities might be is still developing, but already several authors have identified factors that appear to be associated with research translation and the generation of research impact (see Hanney et al. [46], Oliver et al. [47] and Wooding et al. [48] for examples). Some will disagree with a checklist approach. Ward [44] argues that checklists for encouraging research translation are of limited value and that a better mechanism is for researchers to understand the complexity of the translational process and come up with their own bespoke translation activities. Acknowledging that research teams may have resource constraints that limit their ability to develop tailored plans, FAIT provides a checklist of evidence-based translational activities but researchers would be free to add to this list with tailored translational activities and plans.

This study had a number of limitations. Foremost, is that FAIT is a conceptual model and, as yet, untested. Further, the measurement of research impact is not universally welcomed. Critics argue that measurement could have unintended consequences by influencing the direction of research funding with possible adverse effects for blue-sky research [50], where applications for the research outcome are not immediately apparent. However, this problem is not insurmountable. With appropriate time scales and measurement techniques, the prospective measurement of research impact can include the consequences from all research, regardless of whether that research is targeted or blue-sky.

Common to all frameworks that aim to measure research impact, including FAIT, are the following three problems. First, without appropriate study designs it can be difficult to identify causality – did the research cause the impact? Second, it may be difficult to define the extent of attribution – whether the research accounted for all or a small proportion of the impact. Many causes, other than research-generated knowledge, may lead or contribute to an impact. This problem is exacerbated by communication and knowledge sharing, because research and development is now globalised [33, 51]. This worldwide sharing of knowledge makes it contentious to exclude the contribution of global research outputs and to claim a specific research project is fully responsible for a particular impact. The third problem is timing; the impact from research may take more than a decade to materialise. Hanney et al. [46] identify instances where impacts have taken several decades. Depending on the point of measurement, the measurement of impact may fail to capture as yet unrealised benefits [51]. This problem is typically thought to affect basic science discoveries, which might require decades before societal measures of impact are recorded. Addressing this issue would require ongoing updating with a need to gather and report evidence of research translation and impact as it unfolds. Additionally, simulation modelling can be included within SROI where the best available evidence is used to model future impact values.

The changing policy landscape, with respect to the funding of health and medical research, is likely to see increased use of frameworks such as FAIT. In many countries, including Australia, the routine measurement of research impact is becoming embedded across the spectrum of research. The creation of the Australian Government’s Medical Research Futures Fund and its AU$20 billion investment to support health and medical research, will ensure future funding will be partly insulated from changes in the economic cycle. However, this potential increase in security for research funding will be met with heightened expectations that this public investment will deliver greater yields for the community. Key Australian funders are in unison when it comes to statements on the need to measure research impact and increase collaboration and engagement between researchers and end users. However, the need is not just to measure research translation and research impact. The need is for frameworks that incentivise and assist researchers understand, plan for, and implement processes to increase the likelihood of research translation. If research translation is optimised, this increases the chance for research-generated knowledge to generate impact.

The routine use of FAIT in the research community will depend on its ability to provide relevant and robust results and to do so efficiently; that is, to avoid undue burden on researchers with regard to data collection, analysis and reporting. The next step is to evaluate FAIT in a scientific setting and to collect evidence as to its ability to encourage translational activities and behaviours, to assess its effectiveness in reporting research impact, and to report the resources it required for implementation.

## Conclusions

FAIT is a mixed methods approach to encourage and measure research translation and research impact. Its novelty is to add a new aim for measurement activities beyond accountability purposes, that is, to actively encourage research translation to optimise the likelihood of research-generated knowledge being used and, hence, generate impact. FAIT combines methods that bring different perspectives to understanding and measuring research impact. Supported by a program logic model, FAIT combines quantitative and qualitative measurement techniques, with the latter providing an opportunity to explain complex bidirectional translational pathways. Embedded within FAIT is a module for performance monitoring and feedback with the goal of encouraging research translation. The scorecard approach to reporting outcomes and impact maintains simplicity and is a useful communication tool. The inclusion of ‘return on investment’ is a metric that we believe research funders will increasingly require.

## Abbreviations

CBA, Cost-benefit analysis; FAIT, Framework to Assess the Impact of Translational health research; HMRI, Hunter Medical Research Institute; SROI, Social Return on Investment
